# A Novel Non-Invasive Thermometer for Continuous Core Body Temperature: Comparison with Tympanic Temperature in an Acute Stroke Clinical Setting

**DOI:** 10.3390/s22134760

**Published:** 2022-06-23

**Authors:** Miloš Ajčević, Alex Buoite Stella, Giovanni Furlanis, Paola Caruso, Marcello Naccarato, Agostino Accardo, Paolo Manganotti

**Affiliations:** 1 Clinical Unit of Neurology, Department of Medicine, Surgery and Health Sciences, Cattinara University Hospital ASUGI, University of Trieste, Strada di Fiume, 447-34149 Trieste, Italy; majcevic@units.it (M.A.); giovannifurlanis@yahoo.it (G.F.); paolacaruso83@gmail.com (P.C.); marcello.naccarato@asugi.sanita.fvg.it (M.N.); pmanganotti@units.it (P.M.); 2 Department of Engineering and Architecture, University of Trieste, Via A. Valerio, 10-34127 Trieste, Italy; accardo@units.it

**Keywords:** continuous non-invasive temperature monitoring, core temperature, clinical thermometry, acute stroke, heat flux, wearable device

## Abstract

There is a growing research interest in wireless non-invasive solutions for core temperature estimation and their application in clinical settings. This study aimed to investigate the use of a novel wireless non-invasive heat flux-based thermometer in acute stroke patients admitted to a stroke unit and compare the measurements with the currently used infrared (IR) tympanic temperature readings. The study encompassed 30 acute ischemic stroke patients who underwent continuous measurement (Tcore) with the novel wearable non-invasive CORE device. Paired measurements of Tcore and tympanic temperature (Ttym) by using a standard IR-device were performed 3–5 times/day, yielding a total of 305 measurements. The predicted core temperatures (Tcore) were significantly correlated with Ttym (r = 0.89, *p* < 0.001). The comparison of the Tcore and Ttym measurements by Bland–Altman analysis showed a good agreement between them, with a low mean difference of 0.11 ± 0.34 °C, and no proportional bias was observed (B = −0.003, *p* = 0.923). The Tcore measurements correctly predicted the presence or absence of Ttym hyperthermia or fever in 94.1% and 97.4% of cases, respectively. Temperature monitoring with a novel wireless non-invasive heat flux-based thermometer could be a reliable alternative to the Ttym method for assessing core temperature in acute ischemic stroke patients.

## 1. Introduction

Among the physiological parameters commonly monitored in clinical practice, body temperature is one of the major clinical signs and is used to influence clinical management decisions and the diagnosis of certain diseases [1]. Several sites and devices are commonly used to measure body temperature [2,3,4], and due to local metabolism effects, anatomical characteristics, tissue conduction, and blood flow, measurements taken by peripheral thermometers can be highly variable and underestimate the core temperature [5,6,7,8,9]. The inter- and intraindividual variability in normal body temperature further complicate the definition of fever conditions [10], and the most important factors are the site of measurement and the patient’s age [11]. Fever has been defined as a core temperature above 38.3 °C [12]; nevertheless, differences are present between core and peripheral body temperatures [13], and core body temperature is rarely measured in non-surgical or intensive care settings due to the invasiveness of the devices and techniques—e.g., the insertion of vascular, oesophageal, rectal, or bladder catheters [1,14,15]. Fever can be found in about half of patients admitted to intensive care units (ICU) and can be associated with clinical worsening and increased mortality [16,17], and a similar prevalence has been found in patients admitted with stroke [18]. In stroke patients, the continuous monitoring of body temperature is recommended, but it can be often impractical, and, in most cases, it relies on peripheral temperature measurements [19,20].

Due to the great importance of measuring body temperature with non-invasive and accurate methods, novel devices have been developed to measure or estimate core temperature from the skin surface, including the zero-heat-flux thermometers that have been mostly evaluated in surgery and ICU [21]. Other devices have been developed, deriving core body temperature noninvasively from heat flux gradients [22]. Such devices—particularly those that are small and adherent to the patients’ body and do not require wired connections—could be applicable in a wide range of clinical settings, including people with acute neurological diseases. In addition, especially during the COVID-19 pandemic, there was a growing use of forehead temperature measurements in clinical settings as well [23,24,25].

The main aim of the present study was to evaluate the use of a novel wireless non-invasive heat flux-based thermometer in acute stroke patients admitted to a stroke unit and to compare the measurements with the currently used infrared (IR) tympanic measurements. Moreover, the secondary aim was to compare IR forehead temperature readings with tympanic measurements performed at the same time.

## 2. Materials and Methods

An ecologically valid design was used to compare the measurements from the novel device and the practices currently used in the stroke unit. All the patients that were consecutively admitted to the stroke unit from 01.02.2021 to 31.07.2021 with a diagnosis of acute stroke were considered eligible to enter the study. Patients were excluded if they were expected to stay in the stroke unit for less than 3 days, if they were found to be positive for SARS-CoV-2 based on a nasopharyngeal swab at admission, or if they suffered from ear inflammation, forehead swelling, head injuries, or skin or peripheral vascular disease which might have impacted the temperature readings. Participants were also excluded if it was not possible to apply the novel device more than 4 h from admission. Demographics and clinical data were also registered. The device was applied to the left side of the chest according to the manufacturer’s instructions and using the provided adhesive patch. The Bluetooth connection was activated and connected with a tablet for clinical use. The recordings for each participant started from 1 h after the device application and lasted for a maximum of 72 h. All the procedures were performed according to the Declaration of Helsinki, and the local institutional review board and ethics committee (CEUR-FVG) approved the study with an approval number of 115/2018.

### 2.1. Novel Thermometer Characteristics

A novel wireless non-invasive heat flux-based thermometer was used in this study to estimate core body temperature (Tcore). CORE (greenTEG AG, Ruemlang, Switzerland) is a commercially available wearable device that predicts body temperature continuously and non-invasively using the Calera™ technology from greenTEG AG. Body temperature is calculated on the device by an algorithm using built-in heat flux and skin temperature sensors and is transmitted via Bluetooth and ANT communication protocols. The algorithm is based on a combination of classical biophysical and machine learning models trained on more than 1 million data points. In this study, the CORE model 2021-Q1 with firmware 0.4.0 has been used. Compared to the zero-heat-flux method (3 m spot on/bair hugger), no active heating is required, allowing the device to be low-power and autonomous/mobile. In contrast to the double sensor method [26], where the temperature sensors need to be stacked on top of each other, the heat flux and temperature sensors of CORE are placed on the same level. This allows for a thinner and more compact design.

### 2.2. Tympanic and Forehead Temperature

Body temperature was routinely assessed by the healthcare staff—with the use of an IR tympanic thermometer (TEGENIUS3, Tyco Healthcare) and a non-contact IR thermometer (NCIT) (HG01, Comelit)—a minimum of 3 times/day, with more measurements conducted when the patients were found to be hyperthermic (above 37.5 °C tympanic temperature). Tympanic temperature (Ttym) was assessed by measuring temperature two times (10 s between the measurements) from both clean ears by carefully positioning the device and reporting the mean value together with the date and time of measurement. At the same time, forehead skin temperature (Tfor) was assessed with the NCIT from the mid-forehead, approximately 2 cm above the glabella, and by keeping the device at about 10 cm, as indicated by the manufacturer. Two readings were performed, and the mean was reported.

### 2.3. Statistical Analyses

All analyses were performed with MATLAB (The Mathworks Inc.). A Bland–Altman analysis was performed to evaluate the agreement between the novel non-invasive device (CORE) and the commonly used method (Ttym), as well as that between Tfor and Ttym. In the Bland–Altman analysis, the bias that summarizes a degree of agreement between the two methods was calculated as the mean difference between the two methods of measurement. The 95% limits of agreement, which represent where 95% of the future differences between the two methods would be expected, were calculated. The proportional bias was also assessed by performing a linear regression between the difference between the two measures and the mean temperature for each paired measurement. In addition, the correlation between Tcore and Ttym, as well as that between Tfor and Ttym, were investigated. *p*-values < 0.05 were considered statistically significant.

The accuracy of the identification of the presence or absence of hyperthermia and fever by the predicted Tcore, which might be useful in clinical practice, was also investigated. Hyperthermia was defined for temperatures ≥37.5 °C, and fever was defined for temperatures ≥ 38.3 °C, according to the previous literature. Ttym was used as the reference temperature [12,27,28]. To account for the value of continuous temperature monitoring every 1 h, the data were analyzed to report the number of hyperthermic or fever episodes that were not recognized by the tympanic method due to the absence of continuous measurements. A single episode included all the CORE measurements between the first point above the hyperthermia and fever cut-offs and the return to euthermic values.

## 3. Results

The data were collected from a sample of 30 patients (6 females, 71.9 ± 18.9 y, age range 22–97 y) who were admitted with a diagnosis of acute ischemic stroke within 6 h of the onset of the symptoms. The body mass index was higher than 25.0 in 14 (46.6%) patients in the sample. The NIHSS was 7 ± 6, and all the subjects had an anamnestic mRS ≤ 2. Demographics and stroke characteristics are reported in Table 1. The novel CORE device was applied between 30 min and 2 h and 35 min from stroke unit admission, and recorded data were included starting from the second hour of measurement to allow for proper stabilization. Routine body measurements were taken, on average, 3 times/day, with a maximum of 5 times/day. A total of 305 measurements for the Tcore and Ttym recordings were included in the final analyses, while 246 Tfor measurements were collected due to technical issues.

The mean error of the novel wireless non-invasive heat flux-based device compared to the standard tympanic temperature measurements was 0.11 °C. In particular, 85% of all the Tcor measurements were within ±0.5 °C of the tympanic measurements.

**Figure 1:**
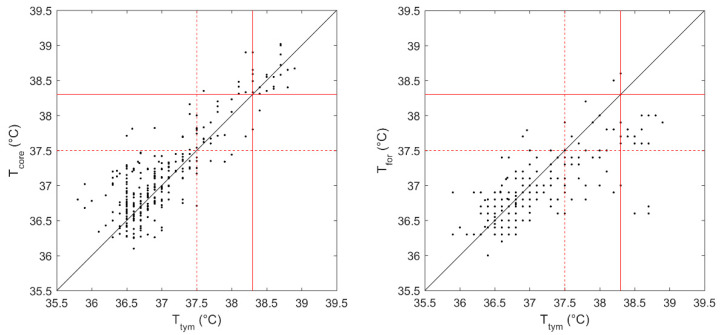
The estimated core temperature (Tcore) (**left panel**) and the forehead temperature (Tfor) (**right panel**)are plotted against the tympanic temperature (Ttym) for each measurement. The red dotted line at a cut-off temperature of 37.5 °C indicates the presence or absence of “hyperthermia”, while the red line at a cut-off value of 38.3 °C indicates the presence of “fever”. The black line suggests the identity with Ttym.

In Figure 1, the estimated core temperatures (Tcore) and the forehead temperatures (Tfor) were plotted against the tympanic temperatures (Ttym) for each measurement. A significant linear correlation was observed between Tcore and Ttym (r = 0.89, *p* < 0.001). Similarly, a moderate correlation was also observed between Tfor and Tymp (r = 0.74, *p* < 0.001).

**Figure 2:**
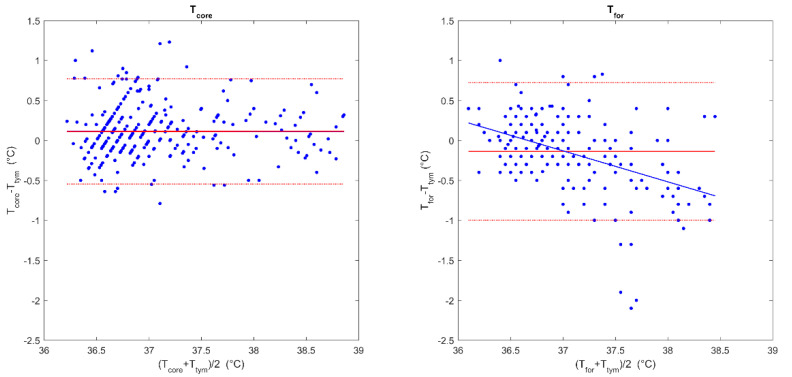
Comparison of the paired temperature measurements. Bland–Altman plots for the estimated core temperature (Tcore) and tympanic temperature (Ttym) (**left panel**) and for the forehead temperature (Tfor) and Ttym (**right panel**). The continuous red line indicates the mean difference between the Tcore or Tfor and Tym, while the red dotted lines indicate 95% confidence intervals. The blue line indicates the regression line between the difference between the two measures and the mean temperature for each paired measurement.

The comparison of the Tcore and Ttym measurements by Bland–Altman analysis (Figure 2; left panel) showed a good agreement between them, with a very low mean difference of 0.11 ± 0.34 °C (95%CI −0.55–0.77 °C). No proportional bias was detected (B = −0.003, *p* = 0.923).

The Bland–Altman comparison between the Tfor and Ttym (Figure 2; right panel) showed a mean difference of −0.14 ± 0.44 °C (95%CI −0.72–1.00 °C). However, a proportional bias was detected (B = −0.388, *p* < 0.001), with a trend of an overestimation of lower temperatures and an underestimation of higher temperatures. Due to the aforementioned trend, an error up to 1.0 °C was possible in the range between 36.0 °C and 38.5 °C.

### Prediction Accuracy of the Presence or Absence of Hyperthermia and Fever

Sixty-five (21.3%) of the Ttym measurements were found to be indicative of hyperthermia, using a cut-off value of 37.5 °C, whereas 25 (8.2%) suggested fever by applying a cut-off of 38.3 °C. When using Tcore, 287 (94.1%) measurements correctly predicted the presence or absence of hyperthermia, and 297 (97.4%) predicted the presence or absence of fever. Tcore suggested false negative hyperthermia in nine measurements (2.9%) and false positive hyperthermia in nine measurements (2.9%), while a false negative fever was found in two measurements (0.6%) and a false positive fever was found in six measurements (2.0%). In contrast, Tfor predicted the presence or absence of hyperthermia in 215 (87.4%) of the measurements and the presence or absence of fever in 222 (90.2%) measurements. Tfor suggested false negative hyperthermia in 26 measurements (10.6%) and false positive hyperthermia in 5 measurements (2.0%), while false negative fever was found in 23 measurements (9.3%) and false positive fever was found in 1 measurement (0.4%) (Figure 2).

In 15/30 patients, at least one hyperthermic or fever episode was not detected by non-continuous measurements with Ttym. In particular, 19 hyperthermic and 2 fever episodes were detected by the CORE continuous measurement and not detected by the standard tympanic method.

## 4. Discussion

The strong desire to monitor human health data is driving the rapid growth of wearable biosensing technologies. Among the different wearable biosensing devices, temperature measurement has proven to be an important physiological parameter [29]. The accurate and continuous measurement of the human core body temperature by a wearable device is of great significance for human healthcare and disease monitoring [30,31]. However, accurate monitoring of body temperature often represents a challenge in clinical practice due to the different devices available, the site of measurement, and the possibility of the healthcare staff obtaining frequent measurements when continuous devices are not available.

This study provides early evidence of the usability of a novel non-invasive wireless heat flux-based thermometer that could provide continuous monitoring and estimation of core body temperature. The estimated core temperatures showed a good agreement (0.11 ± 0.34 °C) and no proportional bias with the tympanic measures, which is a commonly used method in everyday clinical practice in post-stroke patients. The observed mean error compared to the standard tympanic temperature measurements was comparable with that observed in previous clinical studies [6,9], while the calculated limits of agreement were slightly higher than the ±0.5 °C limits that have been used in previous studies in the clinical setting [6,9,32,33,34] and which correspond to the usual magnitude of the human circadian temperature variation [35,36].

Indeed, although such technology might have some limitations in providing an assessment of core body temperature during exercise and its precision should be further evaluated [37,38,39], it might be useful for detecting hyperthermic events in the clinical setting, particularly when compared to other non-invasive methods which might not provide continuous temperature readings [40,41,42] or when patients have to be remotely monitored [1,43,44,45,46]. Our results showed that a novel non-invasive wireless heat flux-based thermometer was able to detect body temperature changes measured by the tympanic thermometer as the presence or absence of hyperthermia (94.1%) and the presence or absence of fever (97.4%) in the post-stroke clinical setting.

In stroke patients, the American Heart Association (AHA)/American Stroke Association (ASA) guidelines recommend obtaining body temperature measurements every 30 min while in the emergency department and every 4 h in the acute setting (stroke unit) [47,48]. However, these guidelines do not define the most appropriate site of measurement, despite the variety of practices ranging from oral, axillary, tympanic, and core body temperature, but recommend caution, as all these methods might differ from each other and might underestimate brain temperature [48]. These differences should be considered when defining the body temperature limits to start cooling and antipyretic treatments, as 37.5 °C is often reported as a cut-off value for worsening stroke symptoms or other declines in neurological status [47].

In this ecologically valid study, the standard clinical practice was maintained by the healthcare staff, meaning they used the standard devices and approaches to measure body temperature. The first finding is represented by the frequency of routinely taken measurements, suggesting that most of the patients were monitored three times per day (usually at 7:00, 14:00, and 22:00), and only in case of fever did they receive additional measurements up to five times per day. This observation suggests that, despite the guidelines recommending more frequent monitoring of body temperature (six times per day), it might be impractical and unfeasible in some clinical settings. The main device used to measure body temperature was the infrared ear thermometer, which represents one of the most common methods to non-invasively assess body temperature by measuring tympanic temperature [49]. Our results also showed the importance of continuous monitoring. Indeed, in fifteen out of thirty patients, at least one possible hyperthermic or fever episode was not detected by non-continuous measurements by the standard tympanic method. The higher measurement frequency allowed by the wearable device detected nineteen hyperthermic and two fever episodes which were not identified by tympanic measurements. Due to the absence of a continuous gold-standard core body temperature measurement, it is not possible to define if those missing events were effective hyperthermic/fever events or false positives; most of the missed events (76%) occurred during the night, when usually 8–10 h passed between the two tympanic temperature measurements.

Among the new devices that have been applied to non-invasively measure body temperature and which became particularly popular during the recent COVID-19 pandemic, non-contact infrared thermometers have been used to measure the surface temperature at the forehead. However, conflicting results have been found in determining its accuracy to predict fever, particularly when non-standardized protocols are adopted (e.g., distance, position, etc.) and the devices are not properly calibrated [8,50,51,52,53,54,55].

Recently, to eliminate the environmental effects and thus improve the accuracy in estimating core body temperature, methods based on direct and correcting temperature data for measurements with an infrared thermal camera [56] and methods based on a dynamic model for measurements with a passive heat flow sensor [57] were proposed. In addition, it has been shown that personalized models can improve measurement accuracy [30].

The forehead is also used as a site of measurements for the zero-heat-flux method and the double sensors method—non-invasive solutions to estimate core body temperature that were shown to be reliable in a wide set of conditions [58,59,60]. Indeed, several active or passive sensing techniques for the non-invasive estimation of core body temperature based on heat flow have been proposed. The zero-heat-flux method [61] is based on active sensors which typically contain a self-heating layer to prevent heat transfer between the skin and the environment. Usually, an active sensing device consists of an insulator patch covered by an electric heater, which creates an isothermal tunnel from the body to the skin surface, allowing for the estimation of the temperature of deeper tissues from the skin temperature. These sensors provide clinicians with the ability to non-invasively monitor body temperature, present a good agreement with core body temperature [62] and sufficient accuracy during stable temperature periods [63,64,65], and are increasingly being used to substitute for more invasive core temperature measurements during surgery and in critical care [21,62]. However, the main disadvantage of the zero-heat-flux method is represented by the presence of a servo-controlled heater, necessary for the heat loss to the ambient atmosphere and requiring considerable alternating current power, therefore making it necessary to have it connected to a socket [59]. If this characteristic minimally influences its application in severe patients requiring prolonged bed rest, in confused or agitated patients, or in moderately severe patients who might be able to stand up and walk (e.g., to go to the bathroom), this might represent a major limitation and prevent its wide use in semi-intensive care units and stroke units. In contrast to the zero-heat-flux method, a novel non-invasive wireless heat flux-based device does not require active heating and is a low-power wireless mobile solution allowing for remote monitoring. Compared to the double sensor method, where the temperature sensors need to be stacked on top of each other, the heat flux and temperature sensor of a novel device are placed on the same level, allowing for a thinner and more compact design and integration into a small non-invasive wearable tool.

This study presents some relevant limitations, which arise from the ecologically valid setting of the study and the maintenance of standard clinical practice. The results were obtained from a relatively small population of only 30 post-stroke patients in a single stroke unit and should be confirmed in a larger study. However, the study encompassed different stroke severity patients, and, on average, every patient was monitored for more than 2.8 days. We also did not observe many temperature readings above 39 °C in order to be able to evaluate the device’s accuracy in a higher temperature range. In addition, the absence of a comparison with a well-validated gold-standard continuous core body temperature measurement, along with the inability to confirm hyperthermic/fever events with other parameters (e.g., blood analyses), suggest considering the results presented in this study as a comparison between devices, and further clinical validation studies, including gold-standard and continuous body temperature measurements, are therefore encouraged.

## 5. Conclusions

In conclusion, temperature monitoring with a novel wireless non-invasive heat flux-based thermometer could be a reliable alternative method for assessing core temperature in acute ischemic stroke patients compared to standard tympanic temperature measurements. The estimated core temperatures showed a good agreement and no proportional bias compared to commonly used tympanic measurements in everyday clinical practice in post-stroke patients, and they provided wireless continuous monitoring in the stroke unit.

## Figures and Tables

**Table 1:** Demographics and stroke characteristics of the included sample. Mean ± sd and proportions, as appropriate.

	Participants (*n* = 30)
***Demographics***	
Age, y	71.9 ± 18.9
Females, *n* (%)	6 (20)
Body mass, kg	69.9 ± 14.9
Body height, m	1.71 ± 9.8
BMI, kg/m^2^	26.3 ± 2.5
***Stroke characteristics***	
NIHSS	7 ± 6
mRS ≤ 2, *n* (%)	30 (100)
Ischemic stroke, *n* (%)	30 (100)

Notes: BMI—Body Mass Index; NIHSS—National Institute of Health Stroke Scale; mRS—modified Rankin Scale.

## Data Availability

Anonymized data are available upon reasonable request to the corresponding author.

## References

[1] Huang F., Magnin C., Brouqui P. (2020). Ingestible Sensors Correlate Closely with Peripheral Temperature Measurements in Febrile Patients. J. Infect..

[2] Vardasca R., Magalhaes C., Marques D., Moreira J., Frade R., Seixas A., Mendes J., Ring F. (2019). Bilateral Assessment of Body Core Temperature through Axillar, Tympanic and Inner Canthi Thermometers in a Young Population. Physiol. Meas..

[3] Chen W. (2019). Thermometry and Interpretation of Body Temperature. Biomed. Eng. Lett..

[4] Childs C. (2018). Body Temperature and Clinical Thermometry. Handb. Clin. Neurol..

[5] Taylor N.A.S., Tipton M.J., Kenny G.P. (2014). Considerations for the Measurement of Core, Skin and Mean Body Temperatures. J. Therm. Biol..

[6] Kimberger O., Thell R., Schuh M., Koch J., Sessler D.I., Kurz A. (2009). Accuracy and Precision of a Novel Non-Invasive Core Thermometer. Br. J. Anaesth..

[7] Pecoraro V., Petri D., Costantino G., Squizzato A., Moja L., Virgili G., Lucenteforte E. (2021). The Diagnostic Accuracy of Digital, Infrared and Mercury-in-Glass Thermometers in Measuring Body Temperature: A Systematic Review and Network Meta-Analysis. Intern. Emerg. Med..

[8] Buoite Stella A., Manganotti P., Furlanis G., Accardo A., Ajčević M. (2020). Return to School in the COVID-19 Era: Considerations for Temperature Measurement. J. Med. Eng. Technol..

[9] Niven D.J., Gaudet J.E., Laupland K.B., Mrklas K.J., Roberts D.J., Stelfox H.T. (2015). Accuracy of Peripheral Thermometers for Estimating Temperature: A Systematic Review and Meta-Analysis. Ann. Intern. Med..

[10] Diamond A., Lye C.T., Prasad D., Abbott D. (2021). One Size Does Not Fit All: Assuming the Same Normal Body Temperature for Everyone Is Not Justified. PLoS ONE.

[11] Geneva I.I., Cuzzo B., Fazili T., Javaid W. (2019). Normal Body Temperature: A Systematic Review. Open Forum Infect. Dis..

[12] O’Grady N.P., Barie P.S., Bartlett J.G., Bleck T., Carroll K., Kalil A.C., Linden P., Maki D.G., Nierman D., Pasculle W. (2008). Guidelines for Evaluation of New Fever in Critically Ill Adult Patients: 2008 Update from the American College of Critical Care Medicine and the Infectious Diseases Society of America. Crit. Care Med..

[13] Bindu B., Bindra A., Rath G. (2017). Temperature Management under General Anesthesia: Compulsion or Option. J. Anaesthesiol. Clin. Pharmacol..

[14] Robinson J.L., Seal R.F., Spady D.W., Joffres M.R. (1998). Comparison of Esophageal, Rectal, Axillary, Bladder, Tympanic, and Pulmonary Artery Temperatures in Children. J. Pediatr..

[15] Lefrant J.-Y., Muller L., de La Coussaye J.E., Benbabaali M., Lebris C., Zeitoun N., Mari C., Saïssi G., Ripart J., Eledjam J.-J. (2003). Temperature Measurement in Intensive Care Patients: Comparison of Urinary Bladder, Oesophageal, Rectal, Axillary, and Inguinal Methods versus Pulmonary Artery Core Method. Intensive Care Med..

[16] Laupland K.B. (2009). Fever in the Critically Ill Medical Patient. Crit. Care Med..

[17] Erkens R., Wernly B., Masyuk M., Muessig J.M., Franz M., Schulze P.C., Lichtenauer M., Kelm M., Jung C. (2020). Admission Body Temperature in Critically Ill Patients as an Independent Risk Predictor for Overall Outcome. Med. Princ. Pract. Int. J. Kuwait Univ. Health Sci. Cent..

[18] Wrotek S.E., Kozak W.E., Hess D.C., Fagan S.C. (2011). Treatment of Fever after Stroke: Conflicting Evidence. Pharmacotherapy.

[19] Kenny T., Barr C., Laver K. (2016). Management of Fever, Hyperglycemia, and Dysphagia in an Acute Stroke Unit. Rehabil. Nurs. Off. J. Assoc. Rehabil. Nurses.

[20] Leys D., Ringelstein E.B., Kaste M., Hacke W. (2007). The Main Components of Stroke Unit Care: Results of a European Expert Survey. Cerebrovasc. Dis..

[21] Conway A., Bittner M., Phan D., Chang K., Kamboj N., Tipton E., Parotto M. (2021). Accuracy and Precision of Zero-Heat-Flux Temperature Measurements with the 3M^TM^ Bair Hugger^TM^ Temperature Monitoring System: A Systematic Review and Meta-Analysis. J. Clin. Monit. Comput..

[22] Mazgaoker S., Ketko I., Yanovich R., Heled Y., Epstein Y. (2017). Measuring Core Body Temperature with a Non-Invasive Sensor. J. Therm. Biol..

[23] Sullivan S., Rinaldi J.E., Hariharan P., Casamento J.P., Baek S., Seay N., Vesnovsky O., Topoleski L. (2021). Clinical evaluation of non-contact infrared thermometers. Sci. Rep..

[24] Hussain A.S., Hussain H.S., Betcher N., Behm R., Cagir B. (2021). Proper use of noncontact infrared thermometry for temperature screening during COVID-19. Sci. Rep..

[25] Lai F., Li X., Wang Q., Luo Y., Wang X., Huang X., Zhang J., Peng J., Wang Q., Fan L. (2022). Reliability of Non-Contact Infrared Thermometers for Fever Screening Under COVID-19. Risk Manag. Healthc. Policy.

[26] Gunga H.-C., Werner A., Stahn A., Steinach M., Schlabs T., Koralewski E., Kunz D., Belavý D.L., Felsenberg D., Sattler F. (2009). The Double Sensor-A Non-Invasive Device to Continuously Monitor Core Temperature in Humans on Earth and in Space. Respir. Physiol. Neurobiol..

[27] Yan F., Zhang D., Xu H., Guo H. (2008). Risk Factors for Fever in Critically Ill Patients with Acute New-Onset Stroke. Neurol. Res..

[28] Leira R., Rodríguez-Yáñez M., Castellanos M., Blanco M., Nombela F., Sobrino T., Lizasoain I., Dávalos A., Castillo J. (2006). Hyperthermia Is a Surrogate Marker of Inflammation-Mediated Cause of Brain Damage in Acute Ischaemic Stroke. J. Intern. Med..

[29] Khan S., Ali S., Khan A., Bermak A. (2021). Wearable Printed Temperature Sensors: Short Review on Latest Advances for Biomedical Applications. IEEE Rev. Biomed. Eng..

[30] Shan C., Hu J., Zou J., Zhang A. (2021). Wearable Personal Core Body Temperature Measurement Considering Individual Differences and Dynamic Tissue Blood Perfusion. IEEE J. Biomed. Health Inform..

[31] Chaglla E.J.S., Celik N., Balachandran W. (2018). Measurement of Core Body Temperature Using Graphene-Inked Infrared Thermopile Sensor. Sensors.

[32] Suleman M.I., Doufas A.G., Akca O., Ducharme M., Sessler D.I. (2002). Insufficiency in a new temporal-artery thermometer for adult and pediatric patients. Anesth. Analg..

[33] Kimberger O., Cohen D., Illievich U., Lenhardt R. (2007). Temporal artery versus bladder thermometry during perioperative and intensive care unit monitoring. Anesth. Analg..

[34] Moran J.L., Peter J.V., Solomon P.J., Grealy B., Smith T., Ashforth W., Wake M., Peake S.L., Peisach A.R. (2007). Tympanic temperature measurements: Are they reliable in the critically ill? A clinical study of measures of agreement. Crit. Care Med..

[35] Sessler D.I., Lee K.A., McGuire J. (1991). Isoflurane anesthesia and circadian temperature cycles in humans. Anesthesiology.

[36] Tayefeh F., Plattner O., Sessler D.I., Ikeda T., Marder D. (1998). Circadian changes in the sweating-to-vasoconstriction interthreshold range. Pflug. Archiv. Eur. J. Physiol..

[37] Tsadok I., Scheinowitz M., Shpitzer S.A., Ketko I., Epstein Y., Yanovich R. (2021). Assessing Rectal Temperature with a Novel Non-Invasive Sensor. J. Therm. Biol..

[38] Moyen N.E., Bapat R.C., Tan B., Hunt L.A., Jay O., Mündel T. (2021). Accuracy of Algorithm to Non-Invasively Predict Core Body Temperature Using the Kenzen Wearable Device. Int. J. Environ. Res. Public Health.

[39] Verdel N., Podlogar T., Ciuha U., Holmberg H.-C., Debevec T., Supej M. (2021). Reliability and Validity of the CORE Sensor to Assess Core Body Temperature during Cycling Exercise. Sensors.

[40] Dakappa P.H., Bhat G.K., Bolumbu G., Rao S.B., Adappa S., Mahabala C. (2016). Comparison of Conventional Mercury Thermometer and Continuous TherCom(^®^) Temperature Recording in Hospitalized Patients. J. Clin. Diagn. Res..

[41] Liu Y., Liu C., Gao M., Wang Y., Bai Y., Xu R., Gong R. (2020). Evaluation of a Wearable Wireless Device with Artificial Intelligence, IThermonitor WT705, for Continuous Temperature Monitoring for Patients in Surgical Wards: A Prospective Comparative Study. BMJ Open.

[42] Verma N., Haji-Abolhassani I., Ganesh S., Vera-Aguilera J., Paludo J., Heitz R., Markovic S.N., Kulig K., Ghoreyshi A. (2021). A Novel Wearable Device for Continuous Temperature Monitoring & Fever Detection. IEEE J. Transl. Eng. Health Med..

[43] Buoite Stella A., Filingeri D., Ravanelli N., Morrison S.A., Ajčević M., Furlanis G., Manganotti P. (2021). Heat Risk Exacerbation Potential for Neurology Patients during the COVID-19 Pandemic and Related Isolation. Int. J. Biometeorol..

[44] Kagiyama N., Hiki M., Matsue Y., Dohi T., Matsuzawa W., Daida H., Minamino T., Kasai T. (2021). Validation of Telemedicine-Based Self-Assessment of Vital Signs for Patients with COVID-19: A Pilot Study. J. Telemed. Telecare.

[45] Ajčević M., Furlanis G., Naccarato M., Caruso P., Polverino P., Marsich A., Accardo A., Manganotti P. (2021). E-Health Solution for Home Patient Telemonitoring in Early Post-Acute TIA/Minor Stroke during COVID-19 Pandemic. Int. J. Med. Inform..

[46] Polverino P., Ajčević M., Catalan M., Bertolotti C., Furlanis G., Marsich A., Buoite Stella A., Accardo A., Manganotti P. (2022). Comprehensive Telemedicine Solution for Remote Monitoring of Parkinson’s Disease Patients with Orthostatic Hypotension during COVID-19 Pandemic. Neurol. Sci. Off. J. Ital. Neurol. Soc. Ital. Soc. Clin. Neurophysiol..

[47] Summers D., Leonard A., Wentworth D., Saver J.L., Simpson J., Spilker J.A., Hock N., Miller E., Mitchell P.H. (2009). Comprehensive Overview of Nursing and Interdisciplinary Care of the Acute Ischemic Stroke Patient: A Scientific Statement from the American Heart Association. Stroke.

[48] Thompson H.J. (2015). Evidence-Base for Fever Interventions Following Stroke. Stroke.

[49] Pusnik I., van der Ham E., Drnovsek J. (2004). IR Ear Thermometers: What Do They Measure and How Do They Comply with the EU Technical Regulation?. Physiol. Meas..

[50] Dell’Isola G.B., Cosentini E., Canale L., Ficco G., Dell’Isola M. (2021). Noncontact Body Temperature Measurement: Uncertainty Evaluation and Screening Decision Rule to Prevent the Spread of COVID-19. Sensors.

[51] Kameda N. (2021). Clinical Accuracy of Non-Contact Forehead Infrared Thermometer and Infrared Tympanic Thermometer in Postoperative Adult Patients: A Comparative Study. J. Perioper. Pract..

[52] Mekjavic I.B., Tipton M.J. (2020). Myths and Methodologies: Degrees of Freedom—Limitations of Infrared Thermographic Screening for Covid-19 and Other Infections. Exp. Physiol..

[53] Mogensen C.B., Wittenhoff L., Fruerhøj G., Hansen S. (2018). Forehead or Ear Temperature Measurement Cannot Replace Rectal Measurements, except for Screening Purposes. BMC Pediatr..

[54] Chen H.Y., Chen A., Chen C. (2020). Investigation of the Impact of Infrared Sensors on Core Body Temperature Monitoring by Comparing Measurement Sites. Sensors.

[55] Dwith Chenna Y.N., Ghassemi P., Pfefer T.J., Casamento J., Wang Q. (2018). Free-Form Deformation Approach for Registration of Visible and Infrared Facial Images in Fever Screening. Sensors.

[56] Silawan N., Kusukame K., Kek K.J., Kuan W.S. A Novel Environment-Invariant Core Body Temperature Estimation for High Sensitivity and Specificity Fever Screening. Proceedings of the 2018 40th Annual International Conference of the IEEE Engineering in Medicine and Biology Society (EMBC).

[57] Atallah L., Ciuhu C., Paulussen I., Bongers E., Blom A., Idrissi A., Noordergraaf G. (2020). Perioperative measurement of core body temperature using an unobtrusive passive heat flow sensor. J. Clin. Monit. Comput..

[58] Teunissen L.P.J., Klewer J., de Haan A., de Koning J.J., Daanen H.A.M. (2011). Non-Invasive Continuous Core Temperature Measurement by Zero Heat Flux. Physiol. Meas..

[59] Sung D.S., Sim S.Y., Jin H.W., Kwon W.Y., Kim K.S., Kim T., Jung Y.S., Ko J.-I., Shin S.M., Suh G.J. (2019). Validation of Non-Invasive Brain Temperature Estimation Models during Swine Therapeutic Hypothermia. Physiol. Meas..

[60] West N., Cooke E., Morse D., Merchant R.N., Görges M. (2020). Zero-Heat-Flux Core Temperature Monitoring System: An Observational Secondary Analysis to Evaluate Agreement with Naso-/Oropharyngeal Probe during Anesthesia. J. Clin. Monit. Comput..

[61] Fox R.H., Solman A.J., Isaacs R., Fry A.J., MacDonald I.C. (1973). A new method for monitoring deep body temperature from the skin surface. Clin. Sci..

[62] Bräuer A., Fazliu A., Perl T., Heise D., Meissner K., Brandes I.F. (2020). Accuracy of zero-heat-flux thermometry and bladder temperature measurement in critically ill patients. Sci. Rep..

[63] Iden T., Horn E.P., Bein B., Böhm R., Beese J., Höcker J. (2015). Intraoperative temperature monitoring with zero heat flux technology (3M SpotOn sensor) in comparison with sublingual and nasopharyngeal temperature: An observational study. Eur. J. Anaesthesiol..

[64] Mäkinen M.T., Pesonen A., Jousela I., Päivärinta J., Poikajärvi S., Albäck A., Salminen U.S., Pesonen E. (2016). Novel Zero-Heat-Flux Deep Body Temperature Measurement in Lower Extremity Vascular and Cardiac Surgery. J. Cardiothorac. Vasc. Anesth..

[65] Atallah L., Bongers E., Lamichhane B., Bambang-Oetomo S. (2016). Unobtrusive monitoring of neonatal brain temperature using a zero-heat-flux sensor matrix. IEEE J. Biomed. Health Inform..

